# Supplementary material for: Application of long read sequencing to determine expressed antigen diversity in Trypanosoma brucei infections
Source: PLoS Negl Trop Dis. 2019 Apr 3;13(4):e0007262. doi: 10.1371/journal.pntd.0007262 (PMC6464242; doi:10.1371/journal.pntd.0007262)
Supplement: S2 Table — (DOCX) [file pntd.0007262.s002.docx]

Plausible mosaic candidates:

| **Day3** | **Day6** | **Day10** | **Day12** |
| --- | --- | --- | --- |
| balbc_3_4/151297/ccs3 (3.5) | balbc_6_0/100673/ccs5 (6.1) | balbc_10_0/102388/ccs3 (10.1) | balbc_12_1/114076/ccs2 (12.1) |
| balbc_3_4/156604/ccs3 (3.5) | balbc_6_1/88751/ccs2 (6.2) | balbc_10_0/118718/ccs6 (10.1) | balbc_12_1/146680/ccs3 (12.1) |
| balbc_3_4/75350/ccs6 (3.5) | balbc_6_2/21985/ccs8 (6.2) | balbc_10_0/128573/ccs2 (10.1) | balbc_12_1/148981/ccs3 (12.1) |
|  | balbc_6_5/151460/ccs7 (6.5) | balbc_10_0/48011/ccs2 (10.1) | balbc_12_1/30571/ccs9 (12.1) |
|  | balbc_6_5/78761/ccs13 (6.5) | balbc_10_0/6845/ccs4 (10.1) | balbc_12_1/40149/ccs2 (12.1) |
|  | balbc_6_5/86021/ccs7 (6.5) | balbc_10_2/138490/ccs5 (10.3) | balbc_12_1/48081/ccs4 (12.1) |
|  |  | balbc_10_4/106314/ccs2 (10.4) | balbc_12_1/9710/ccs9 (12.1) |
|  |  | balbc_10_4/13775/ccs2 (10.4) | balbc_12_1/97157/ccs2 (12.1) |
|  |  | balbc_10_4/82822/ccs2 (10.4) | balbc_12_2/126314/ccs8 (12.2) |
|  |  | balbc_10_5/110598/ccs2 (10.5) | balbc_12_2/90072/ccs7 (12.2) |
|  |  | balbc_10_5/125683/ccs15 (10.5) | balbc_12_3/102971/ccs2 (12.3) |
|  |  | balbc_10_5/14682/ccs9 (10.5) | balbc_12_3/105707/ccs7 (12.3) |
|  |  | balbc_10_5/26181/ccs4 (10.5) | balbc_12_3/128221/ccs3 (12.3) |
|  |  |  | balbc_12_3/156428/ccs2 (12.3) |
|  |  |  | balbc_12_3/40832/ccs2 (12.3) |
|  |  |  | balbc_12_3/50618/ccs4 (12.3) |
|  |  |  | balbc_12_3/67024/ccs3 (12.3) |
|  |  |  | balbc_12_3/75428/ccs8 (12.3) |
|  |  |  | balbc_12_3/83905/ccs2 (12.3) |
|  |  |  | balbc_12_5/135299/ccs5 (12.5) |
|  |  |  | balbc_12_5/145884/ccs2 (12.5) |
|  |  |  | balbc_12_5/40253/ccs3 (12.5) |
|  |  |  | balbc_12_5/46204/ccs6 (12.5) |

*The coloured cells represent identical sequences
