# Supplementary figures and images for: Application of long read sequencing to determine expressed antigen diversity in Trypanosoma brucei infections
Source: PLoS Negl Trop Dis. 2019 Apr 3;13(4):e0007262. doi: 10.1371/journal.pntd.0007262 (PMC6464242; doi:10.1371/journal.pntd.0007262)

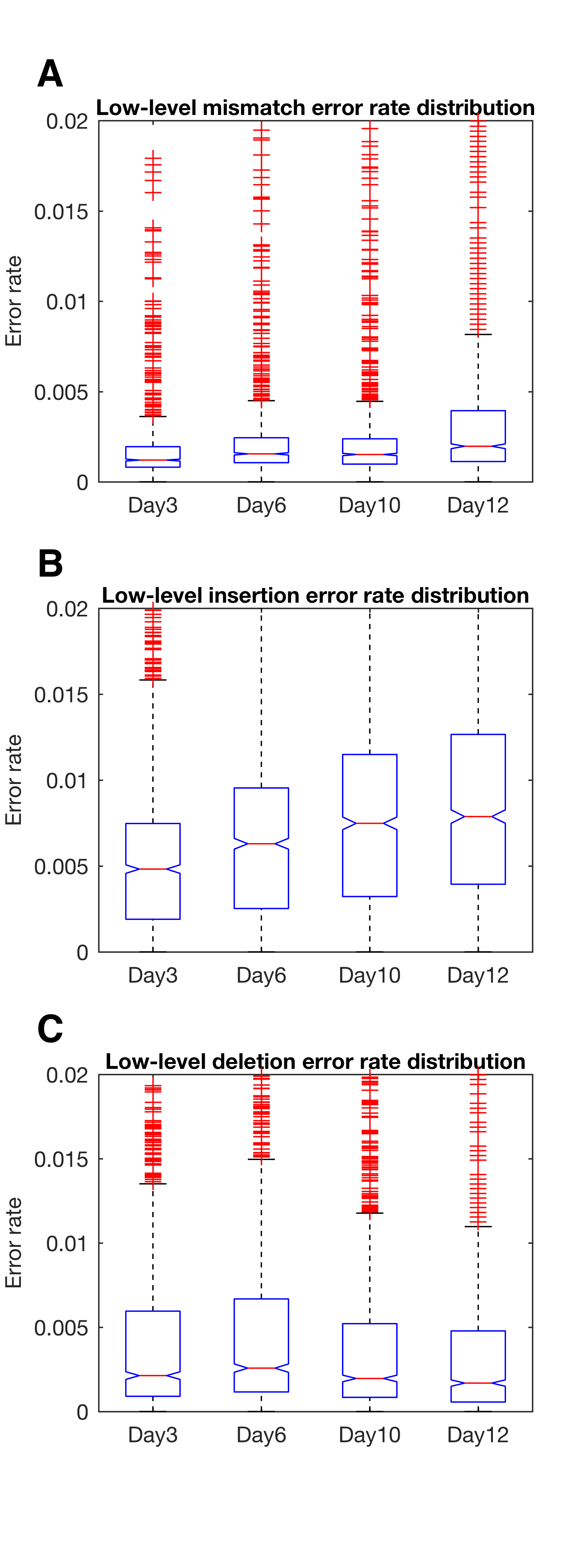

Supplement: S1 Fig — Error (mutation) rate distribution over time (day 3, 6, 10 and 12 post-infection) for reads aligning to VSG Tb08.27P2.380 for (A) mismatches, (B) insertions and (C) deletions; each defined as differences relative to the reference genome sequence of Tb08.27P2.380. For each mutation class and timepoint, the boxplot shows median values and 25th and 75th percentiles, whiskers extend to data extremes, and data outliers are plotted individually (red plus symbols). (TIF) [file pntd.0007262.s003.tif]

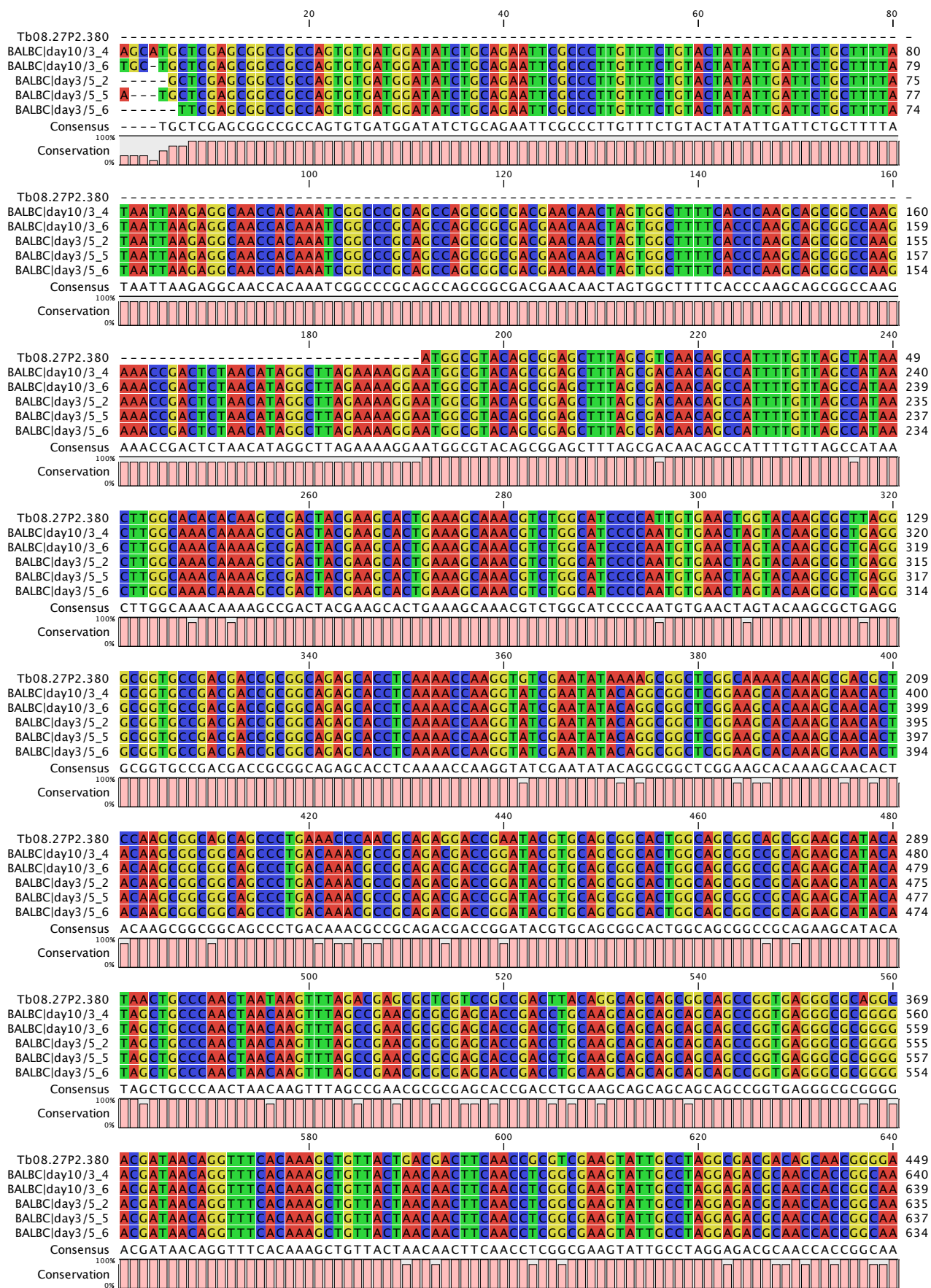

Sample 1 to 5

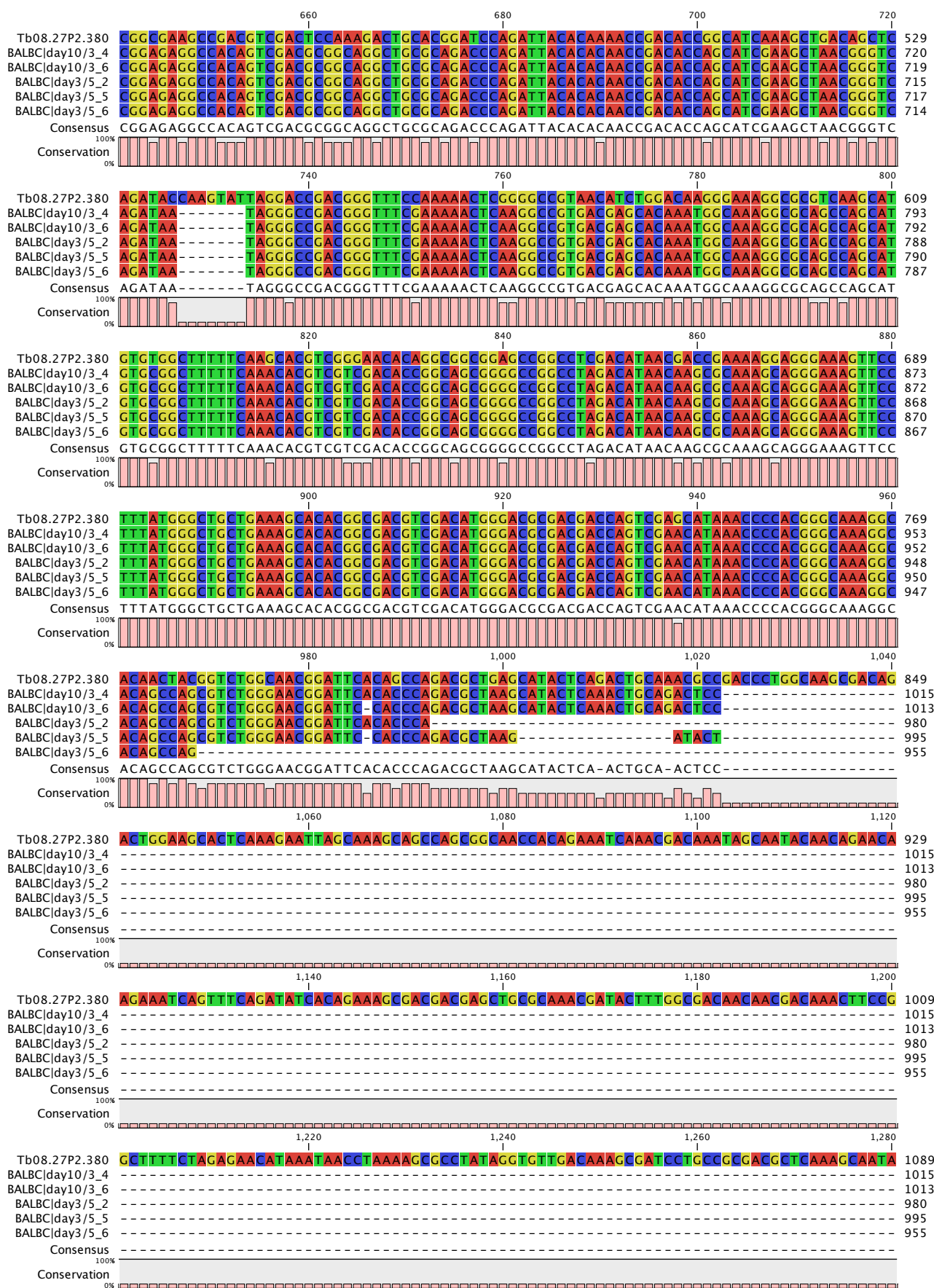

Sample 1 to 5

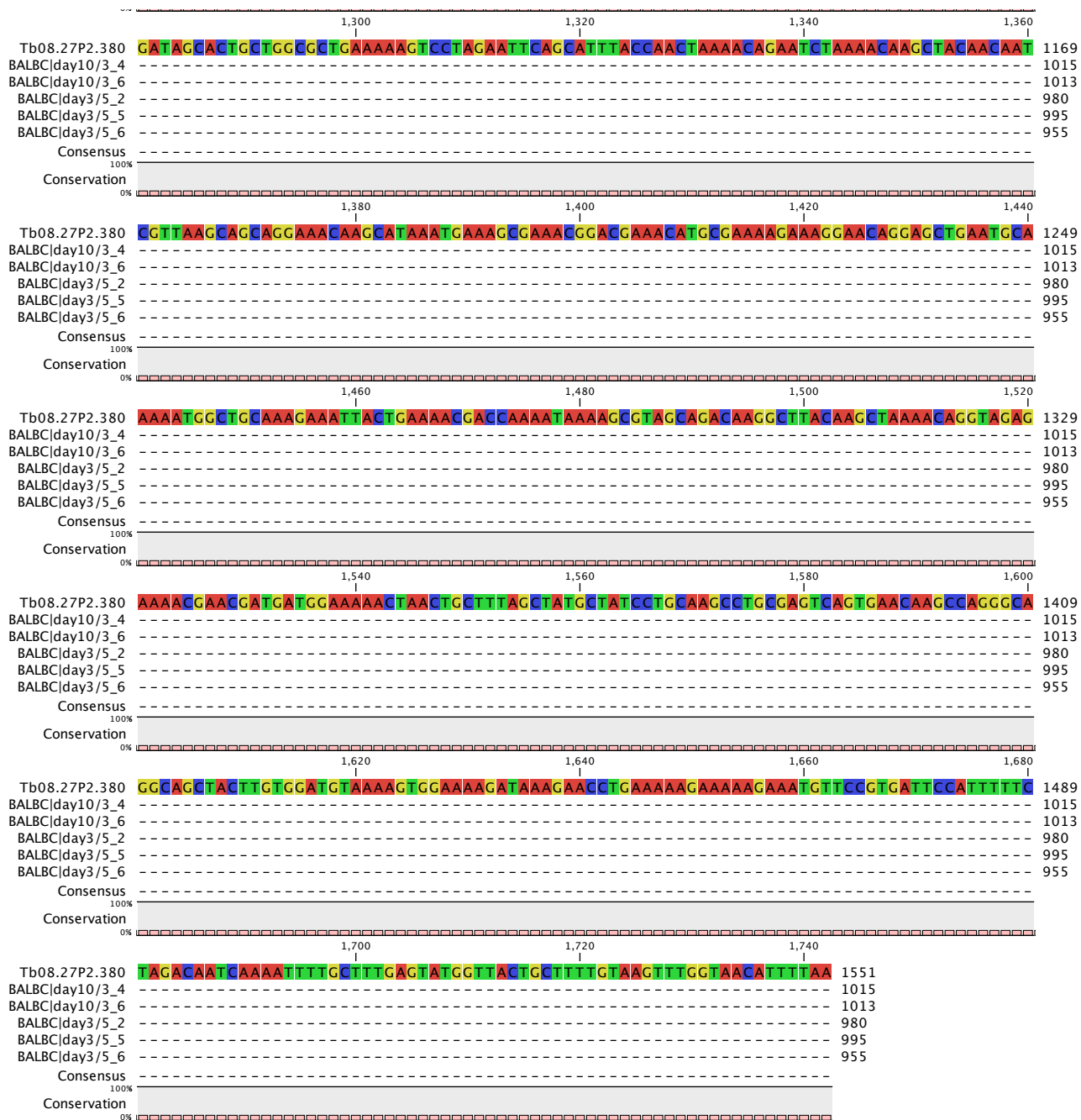

Sample 1 to 5

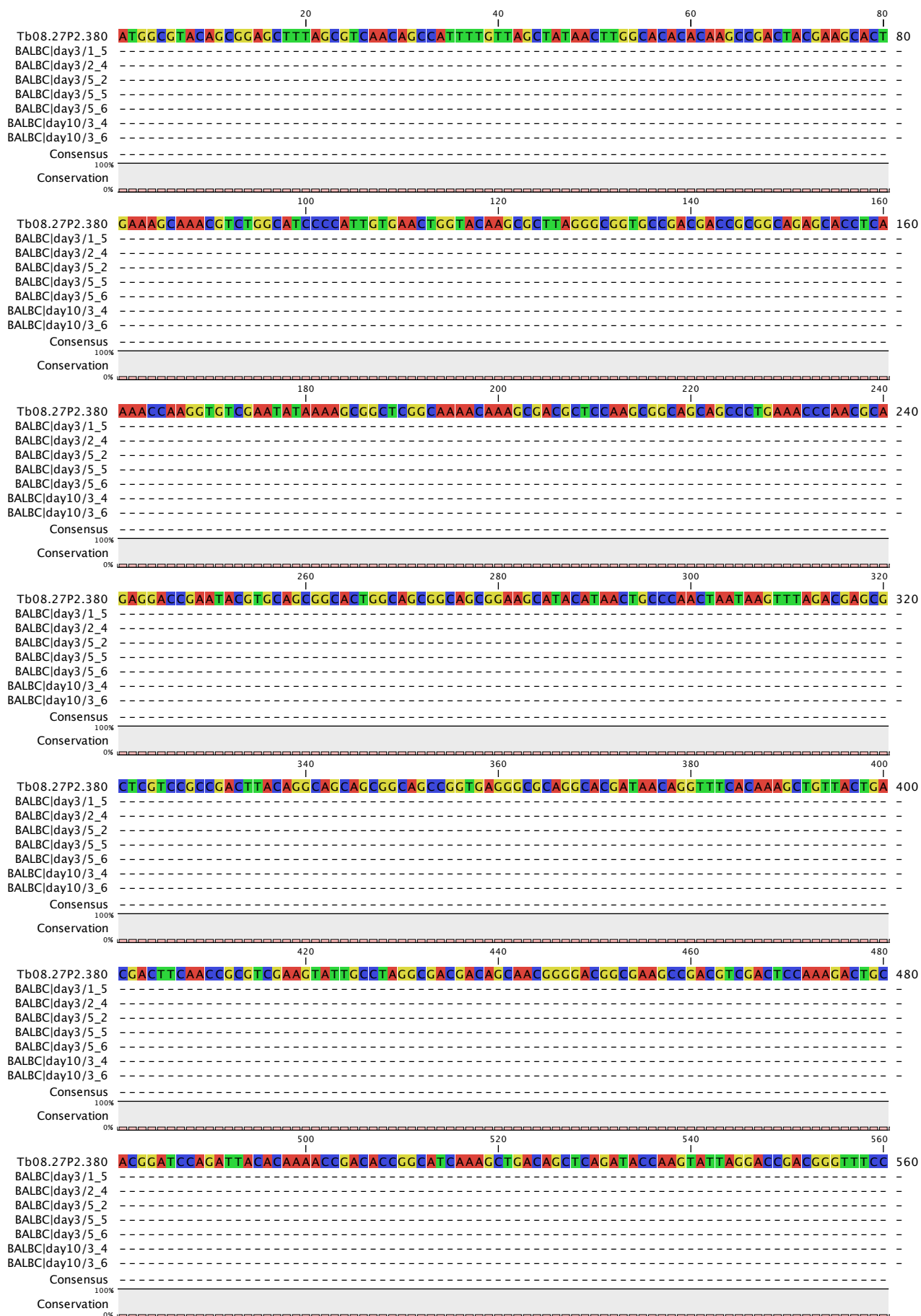

Sample 6 to 12

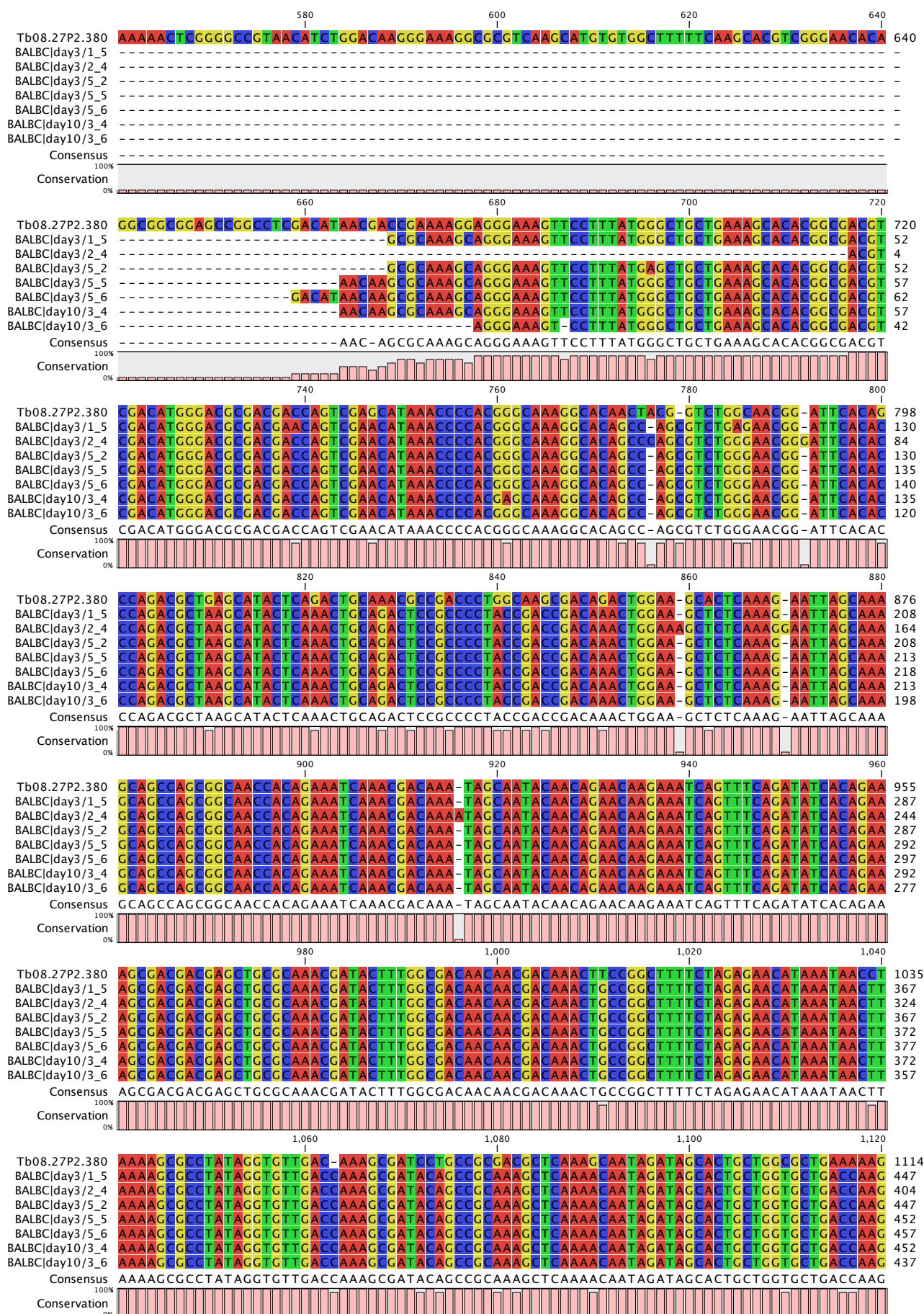

Sample 6 to 12

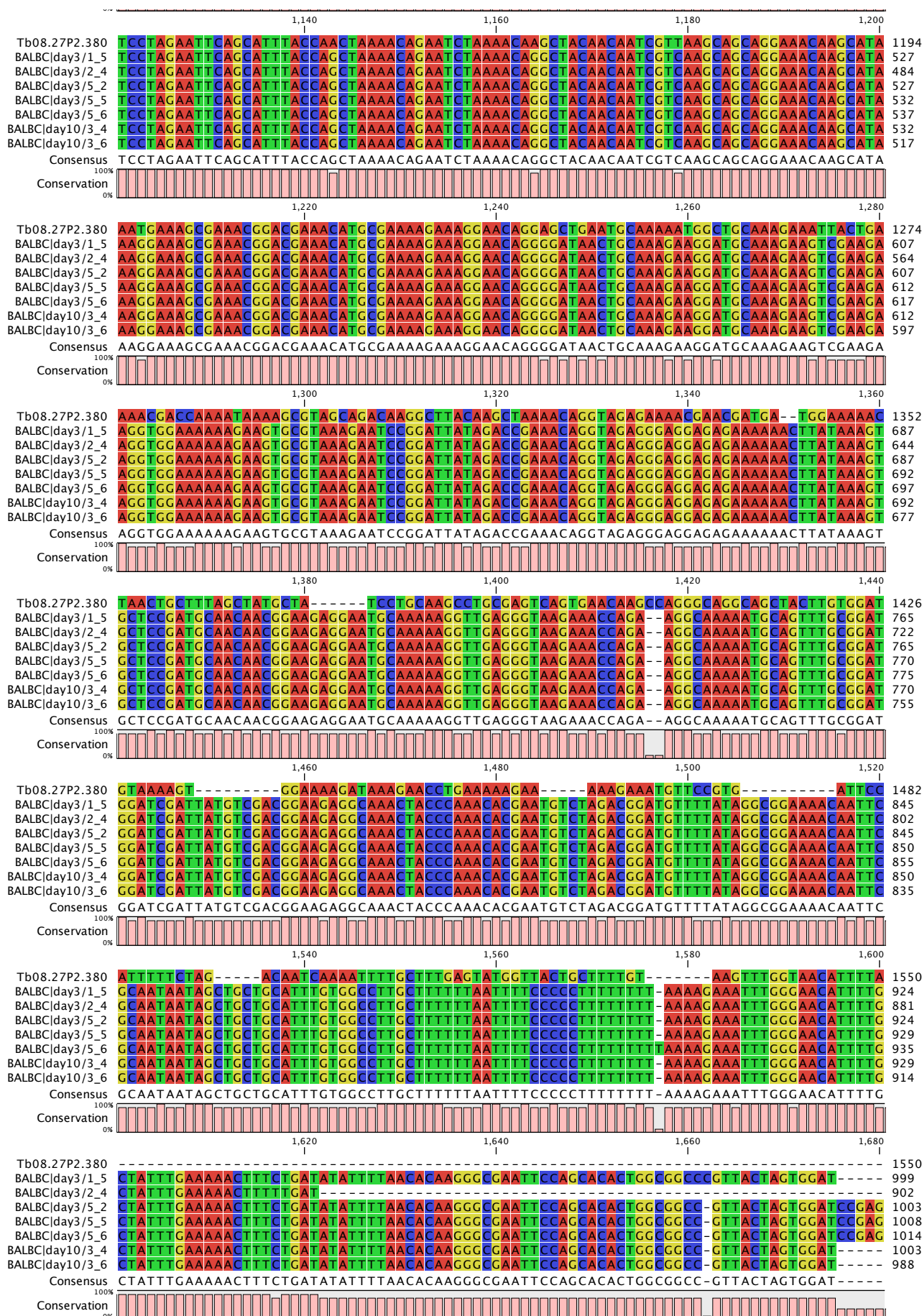

Sample 6 to 12

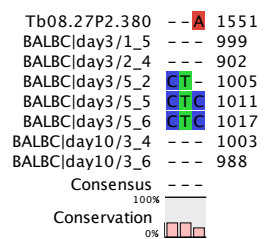

Sample 6 to 12

Supplement: S2 Fig — (PDF) [file pntd.0007262.s004.pdf]

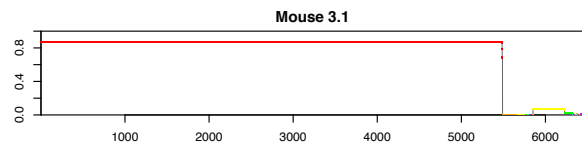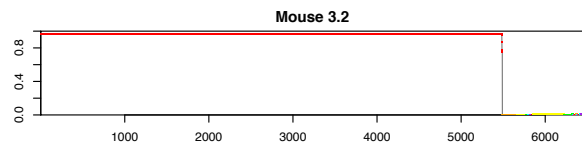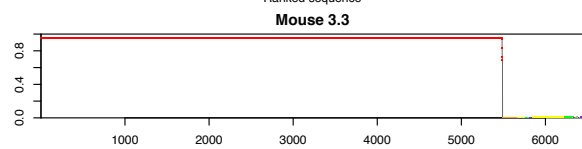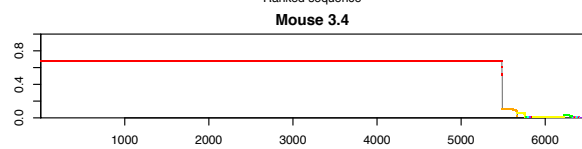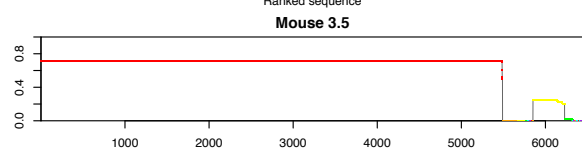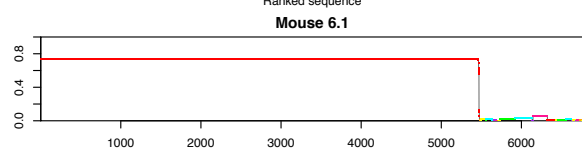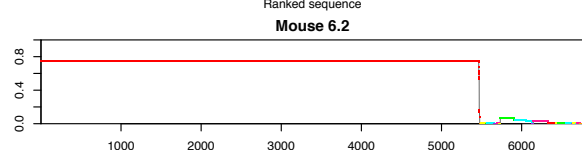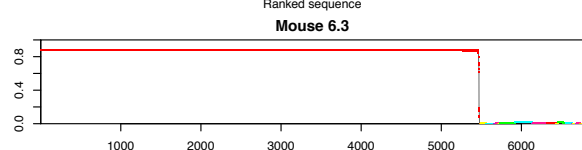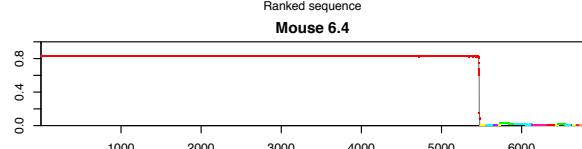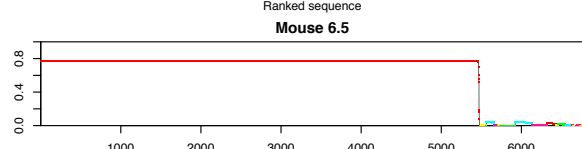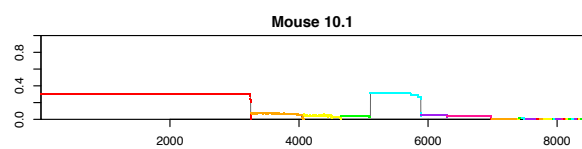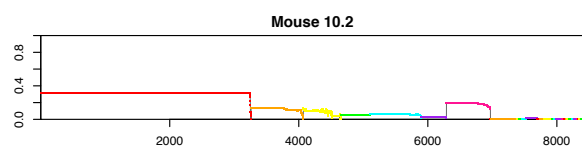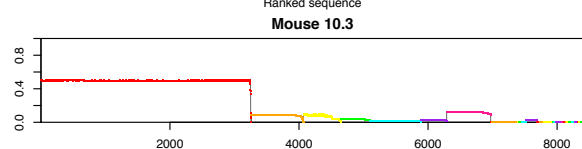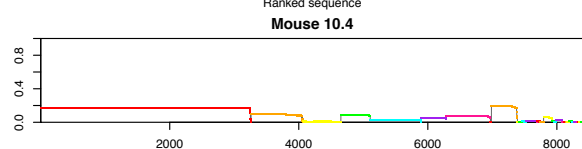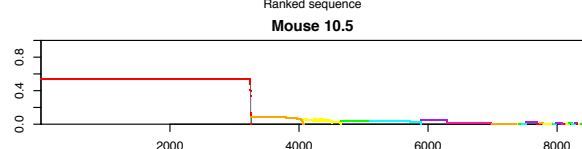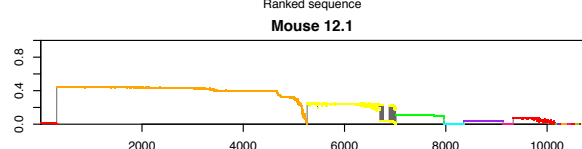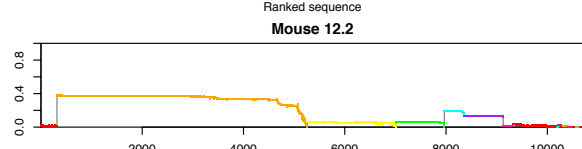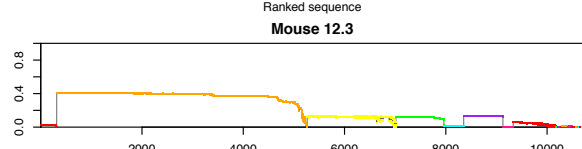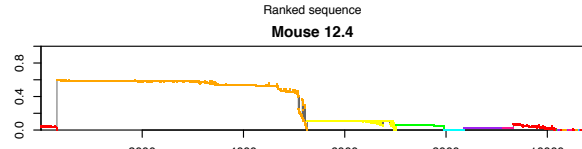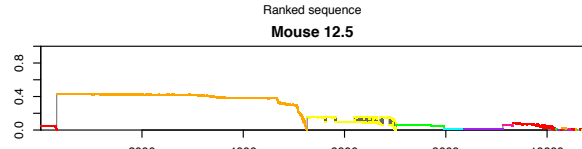

Supplement: S4 Fig — The y-axis indicates how common the cluster is in that mouse and the x-axis indicates how many sequences fall within that cluster. Clusters are colour coded such that a red cluster in mouse 3.1 is defined by the same centroid and clustering threshold as the red cluster in mouse 10.5 etc. (PDF) [file pntd.0007262.s006.pdf]
