# Supplementary material for: Application of long read sequencing to determine expressed antigen diversity in Trypanosoma brucei infections
Source: PLoS Negl Trop Dis. 2019 Apr 3;13(4):e0007262. doi: 10.1371/journal.pntd.0007262 (PMC6464242; doi:10.1371/journal.pntd.0007262)
Supplement: S1 Appendix — (DOCX) [file pntd.0007262.s007.docx]

**S5 Appendix. Clustering algorithm and Diversity analysis detailed methods.**

**Clustering algorithm**

Performing a non-dynamics clustering analysis, using a fixed standard 6% threshold for intra-cluster dissimilarity we observed that most sequences fell into clusters, but there was a large number (~6000) of unclustered reads many of which were only just outside the 6% threshold for a cluster and so should have been assigned to that cluster. To address this a dynamic clustering algorithm was designed which would allow the cluster threshold to grow for each cluster independently allowing us to correctly deal with the unclustered reads. The algorithm proceeded as follows:

1. We used Clustal Omega to calculate genetic distances between each pair of sequences [[45](#_ENREF_45)] and converted these into similarities using a 6% threshold for dissimilarity. All sequence pairs below the threshold for dissimilarity were given a similarity of 1 and those above given 0. This strict cut-off was chosen to allow our method to be comparable to traditional clustering approaches.
2. Centroid sequences that would start off each new cluster were chosen using the core measure from the diversity framework, ordinariness [[46](#_ENREF_46)]. Initial ordinariness O_1i_ of sequence i is the average similarity of any sequence to sequence i, or here the number of sequences within 6% sequence dissimilarity of sequence i. The sequence with the highest ordinariness was chosen as the centroid of the first cluster, and all sequences within 6% genetic distance were included in that cluster.
3. Ordinarinesses, O_ci_, were then recalculated for cluster c (c= 2, 3 …) from all of the remaining sequences not currently allocated to a cluster and the method for identifying a new centroid (step 2) was repeated.
4. From the third cluster onwards (c > 2), however, any sequence with a higher initial ordinariness O_1i_ than the previous cluster centroid must have derived that ordinariness from similarity to an earlier cluster (as otherwise it would have been selected earlier), and is therefore assigned to its closest cluster rather than forming a new cluster. The cluster threshold for that closest cluster is then re-calculated to accommodate the new sequence, and any other sequences that now fall within the new sequence dissimilarity threshold for that cluster are added to it.
5. The clustering algorithm repeats until no more sequences are present or the cluster sizes grow too large and begin to overlap, leaving residual unique (unclustered) sequences.

**SI Diversity analysis**

For the diversity analysis we need to define the *metacommunity* which is the VSG profile on a given day when we pool the data from all the mice from that day (SI Figure 1A). The *subcommunity* is then the VSG profile for a particular mouse on that day. So each metacommunity (day) is made up of 5 subcommunities (mice). By *VSG profile* we mean the distribution of VSGs as illustrated by the pie charts in SI Figure 1A.

When looking at how different each mouse’s VSG profile is within each day one might start by asking: of all the VSGs seen on that day how many are seen in mouse i from that day? If each mouse has half of the possible VSGs from that day then we say there are effectively 2 distinct VSG profiles on that day because this is the minimum number of distinct VSG profiles we would need to account for all the diversity we see, because any given profile only contains half of that day’s diversity. This number of distinct profiles is referred to as *normalised beta diversity* for q=0, and is calculated for each mouse on a given day (see SI Figure 1B). The parameter q weighs the extent to which the relative proportion of each VSG is taken into account when we assess normalised beta diversity. When q=0 we ignore the relative proportions of each VSG, we only consider if each VSG is present or not. As q increases we no longer simply care about how many of the VSGs are present in each mouse, but also how faithfully the mouse (subcommunity) preserves the proportions of each VSGs observed across all the mice from that day (metacommunity). Unless the VSGs are evenly distributed across each mouse we find that increasing q results in an increase in the normalised beta diversity as more VSG profiles are needed to make up for the unevenness in the proportions of VSGs.

We see that for our dataset normalised beta diversity, or the effective number of distinct VSG profiles is close to 1 for q=0 on each day (SI Figure 2B). This is because each mouse on a given day expresses at least some of almost every VSG present on that day, so there is only one VSG profile when we ignore the role of relative abundances of the VSGs. However, as q increases, and we start to care about accuracy of the abundances in the profiles and we are able to detect that some of the mice become increasingly divergent from the rest (e.g. mouse 3.4 and 3.5 in day 3), because they have some VSGs that are more common when compared to the rest of the mice in that day. This is contrast to day 6 where most mice (except for mouse 6.5) broadly agree on not only which VSGs are present (q=0), but also on how common those VSGs are on that day (shown by the closeness of the lines in SI Figure 2B, day 6 for q>0). Day 10 has the most distinct VSG profiles, with a lot of variability between the mice, while on day 12 the mice begin to express similar VSG profiles again (though mouse 12.2 is distinct).

These results are further supported by additional clustering analysis. We can apply the clustering algorithm to each mouse individually (SI Figure 2). Looking at day 3 we see that the orange and yellow clusters only appear in 3 of the mice on day 3 (mice 3.1, 3.4, 3.5), highlighting the diversity between the mice that we saw from the normalised beta diversity analysis. Similarly, the day 10 mice show a lot of variation in clusters of VSGs they express, whereas on day 12 the clustering is broadly the same for each mouse.
